# Physio-Biochemical Insights into the Cold Resistance Variations among Nectarine (*Prunus persica* (L.) Batsch var. *nectarina*) Cultivars

**DOI:** 10.3390/biology13040222

**Published:** 2024-03-28

**Authors:** Guojie Qin, Yifan Liu, Jianzi Liu, Gefang Bian, Shikai Zhang, Yi Liu, Lixiang Zuo, Chunzhen Cheng

**Affiliations:** College of Horticulture, Shanxi Agricultural University, Jinzhong 030801, China; z20213194@stu.sxau.edu.cn (Y.L.); z20213250@stu.sxau.edu.cn (J.L.); z20223343@stu.sxau.edu.cn (G.B.); z20223311@stu.sxau.edu.cn (S.Z.); sxndliuyi@sxau.edu.cn (Y.L.); zuolx@sxau.edu.cn (L.Z.)

**Keywords:** nectarine, cold resistance evaluation, relative water content, osmoregulatory substances, antioxidant enzyme activity, phytohormone accumulation

## Abstract

**Simple Summary:**

In this study, the cold resistance of five main nectarine (*Prunus persica* (L.) Batsch, var nectarine) cultivars grown in Shanxi Province of China was evaluated and compared. To explore the physio-biochemical mechanism underlying their cold resistance variations, semi-lethal low temperature (LT_50_) and thirteen cold resistance related parameters at six timepoints during overwintering were determined and analyzed. Results showed that these five nectarine cultivars could be classified into high cold resistant (HR), moderate cold resistant (MR) and low cold resistant (LR) groups. The HR cultivars were of higher relative water (RW) and proline (PRO) contents and superoxide dismutase (SOD) activity, but much lower relative electric conductivity (RE), malondialdehyde (MDA) content and catalase (CAT) activity. Moreover, the increase of PRO and abscisic acid (ABA) contents and decrease of RW content in HR cultivars were greater than other cultivars. Additionally, redundancy analysis (RDA) revealed that these indexes were significantly correlated with the HR cultivars, indicating that they could be used as potential markers for the cold resistance evaluation of nectarine germplasm resources.

**Abstract:**

Cold stress occurs in late winter and early spring threatens greatly the nectarine industry. In this study, the semi-lethal low temperature (LT_50_) and thirteen cold resistance related parameters of five nectarine cultivars, including ‘Nonglehong little princess’ (LP), ‘Luyou No. 5’ (LY), ‘Nonglehong No. 6’ (NL), ‘Zhongyou No. 20’ (ZY) and ‘Qiuhongzhu’ (QH), were determined. Based on these parameters, they were categorized into high—(HR, including NL and LP), moderate—(MR, including QH) and low-cold resistant (LR, including ZY and LY) groups. The relative water (RW), proline (PRO), soluble sucrose (SS) and soluble protein (SP) contents, and superoxide dismutase (SOD) and peroxidase (POD) activities of HR cultivars were higher while their relative electronic conductivity (RE), malondialdehyde (MDA) and gibberellin acid (GA_3_) contents and catalase (CAT) activity were lower than other cultivars during natural overwintering. Redundancy analysis revealed that the lowest temperature in a day (LT) and LT_50_ significantly explains 69.8% and 10.9% of these physiological variables, respectively. Moreover, GA_3_ and indoleacetic acid (IAA) contents and CAT activity were positively correlated, while PRO, SS, ABA and RW contents were negatively correlated with both LT and LT_50_. Our study will be helpful in understanding the cold resistance variations of nectarine germplasm resources.

## 1. Introduction

Fruit-producing crops, mostly belong to the third most economically important plant family Rosaceae [1], are mainly grown in temperate regions [2]. Although they require chilling to develop fruiting buds during winter [3], the frost occurred in late winter and early spring can severely damage buds, flowers, and fruits, which will lead to production reduction and even whole plant death [2,4]. Therefore, low temperature (cold) stress is regarded as one of the main limiting environmental factors threatening the healthy development of fruit crops industry [5,6].

To combat cold stress, fruit crops have evolved several adaptive mechanisms via various physical and biochemical strategies [5,6]. However, the strategies taken by different plant species varied a lot. Therefore, understanding of the mechanism involved in the cold stress response of certain fruit crop is important and necessary [7]. For the evaluation of cold resistance of plant species, cold damaged symptoms, and changes of photosynthesis and chlorophyll fluorescence related parameters were frequently studied and compared [8]. Osmoregulatory substances and antioxidant enzymes function greatly in balancing the generation and scavenging of reactive oxygen species (ROS) and play key roles in the low temperature adaptive defense responses of plants [9]. Moreover, cold stress can impair cell membrane permeability, disturb ionic and phytohormone homeostasis, and induce electrolyte leakage (EL) [10]. Therefore, the activities of antioxidant enzymes (such as superoxide dismutase (SOD), peroxidase (POD) and catalase (CAT)) [11], accumulations of osmoregulatory substances (including proline (PRO), soluble sugar (SS), soluble protein (SP) and so on), contents of the marker of cell membrane damage malondialdehyde (MDA)) [12] and endogenous phytohormones (such as gibberellin acid (GA_3_), indoleacetic acid (IAA) and abscisic acid (ABA)) [13,14] and EL are often used as important indexes for plant cold resistance evaluations [15,16,17].

The nectarine (*Prunus persica* (L.) Batsch, var nectarine), an important fruit tree belonging to the Rosaceae family, is native to China and is named for its peach-like fruit but with smooth skin. The nectarine fruits are not only popular fresh fruits but can be processed into fruit cans and juice, making their economic value very high [18,19,20]. At present, nectarine is widely cultivated in temperate areas of China, Italy, Span, USA and some other countries [21,22]. Of these nectarine cultivating countries, China has the highest cultivation area and the largest annual output of nectarine in the world. In China, nectarine is mainly cultivated in Shandong, Henan, Shanxi, Hebei and Liaoning provinces, and the annual nectarine fruit output in these provinces accounted for approximately 70% of the total output in China. However, in these provinces (and especially in the high-altitude areas in Shanxi province), cold stresses occur frequently in the late winter and early spring (the key timepoint for the flower bud development) [23], thus influencing greatly the production and quality of nectarine fruits. Therefore, comprehensive cold resistance evaluation of nectarine germplasm resources, screening of cold resistant varieties and clarification of the underlying mechanism of cold resistance are of great importance for the healthy development of nectarine industry. 

At present, there are only a few studies on the cold resistance evaluation and cold resistance mechanism exploration of *P*. *persica* [24], but there are no relevant reports on nectarine. In this study, we first determined the semi-lethal low temperature (LT_50_, the lowest lethal temperatures of 50% individuals) and investigated the field cold resistance of five main nectarine cultivars (‘Nonglehong little princess’ (LP), ‘Luyou No. 5’ (LY), ‘Nonglehong No. 6’ (NL), ‘Zhongyou No. 20’ (ZY) and ‘Qiuhongzhu’ (QH) grown in Shanxi Province. To explore the physio-biochemical mechanisms underlying their cold resistance, thirteen cold resistance related parameters (including relative electric conductivity (RE), contents of relative water content (RW), PRO, MDA, SS, SP, GA_3_, IAA and ABA, activities of SOD, POD and CAT, and ABA/GA_3_) in shoots of these nectarine cultivars at six different timepoints during the late winter and early spring were measured and compared. Then, principal component analysis (PCA) and redundancy analysis (RDA) were performed to evaluate the correlation and contributions of these indexes to the cold resistance of nectarine cultivars. The results obtained in this study will be helpful in the future high cold resistant variety selection and breeding and can provide a basis for the comprehensive evaluation of cold resistance of nectarine germplasm resources. 

## 2. Materials and Methods

### 2.1. Plant Materials

**Figure 1:**
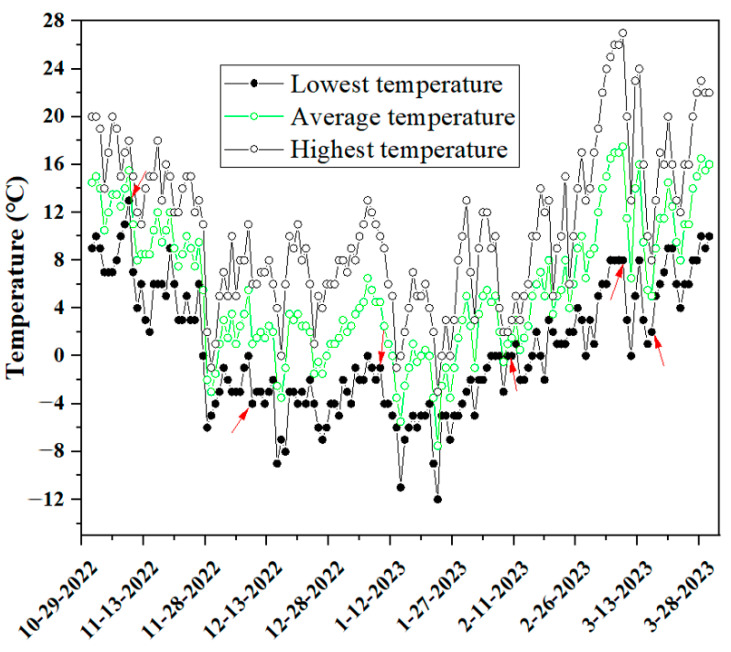
Temperature changes in the nectarine orchard we studied during the natural overwintering. Arrows represent the six timepoints for sample harvesting and determinations of cold resistance related parameters in this study.

Uniform three-year old ‘Nonglehong little princess’ (LP), ‘Luyou No. 5’ (LY), ‘Nonglehong No. 6’ (NL), ‘Zhongyou No. 20’ (ZY) and ‘Qiuhongzhu’ (QH) nectarine trees used in this study were grown in an orchard located in Fengkou Village, Pinglu County, Yuncheng City, Shanxi Province, China (111°9′24″ E, 34°58′1″ N). This region is located in northern warm temperate zone with a continental semi-arid monsoon climate. The frost period for this area is from late October to early April. Fifteen unique and healthy shoots of each nectarine cultivar were randomly selected and picked from the middle of the crown of nectarine plants at six timepoints during overwintering, i.e., 10 November 2022 (T1), 10 December 2022 (T2), 10 January 2023 (T3), 10 February 2023 (T4), 10 March 2023 (T5) and 17 March 2023 (T6). Then, samples were taken back to lab, washed with distilled water and used for further studies. The highest temperature (HT), average temperature (AT) and lowest temperature (LT) at T1–T6 was 18 °C, 6 °C, 10 °C, 2 °C, 27 °C and 8 °C, 15.5 °C, 1 °C, 4.5 °C, 1 °C, 17.5 °C and 5 °C, and 13 °C, −4 °C, −1 °C, 0 °C, 8 °C and 2 °C (Figure 1), respectively. The obvious temperature drop at T6 was caused by the sudden snowfall in the previous day.

### 2.2. Determination of Semi-Lethal Low Temperature (LT_50_)

In March 2023, sampled shoots were divided into six groups, and separately placed in a high and low temperature alternating test chamber (Guangzhou Xingtuo environmental experimental equipment Technology Company, Guangzhou, China) for low temperature treatment at a cooling rate of 4 °C/h to 0 °C (control), −10 °C, −15 °C, −20 °C, −25 °C and −30 °C [11,12], respectively. After treatment for 24 h, shoots were picked out, kept at room temperature for 2 h, and then the middle parts of shoots (excluding flower and leaf buds) were subjected to the determination of electric conductivity. The LT_50_ of each nectarine cultivar was calculated using the formula LT_50_ = ln a/b by combining the relative electric conductivity method with the Logistic equation (y = k/(1 + ae^−bx^)) [25], where y represents the relative electric conductivity, k = 100, a and b are the equation parameters, and x is the treatment temperature.

### 2.3. Cold Injury Investigation and Cold Resistance Classification of Nectarine Cultivars in Field

For each nectarine cultivar, a total of 30 trees were selected to investigate the cold injuries during November 2022 to April 2023. According to the observed cold symptoms, all of these investigated trees were categorized into four grades [26]. Grade 0: No cold injury occurred in branches and branches; Grade 1: Slight injuries appeared in the trunk and branches; Grade 2: Frostbite appeared in branches and small frost crack(s) in the trunk; Grade 3: Most branches were frozen to death, and obvious cracks appeared in the trunk with colloid outflow. Then, the freezing damage index of each cultivar was calculated using the following formula [27]: Freezing damage index = (1 × S1 + 2 × S2 + 3 × S3 + 4 × S4)/(total number of investigated plants × 4), where S1, S2, S3 and S4 represents the number of cold damaged plants belonging to Grade 0~3, respectively. 

### 2.4. Determination of Relative Water Content (RW) and Relative Electrolytic Conductivity (RE)

After removing bud eyes, shoots were cut into small sections. Then, 1 g (M1) shoots were weighed and put into a 60 °C oven (Ningbo Ledian Instrument Manufacturing Company, Ningbo, China) for green removing for 15 min, and dried to constant weight (M2). Finally, the relative water content (RE) was calculated using the formula: RW = M2/M1 × 100%. The RE in nectarine shoots was determined by using the immersion method [28]. 

### 2.5. Determinations of Osmoregulatory Substances Contents, Antioxidant Enzymes Activities and Phytohormones Contents

For the determinations of osmoregulatory substance contents, antioxidant enzymes activities and phytohormones contents, the middle part to shoots (excluding flower and leaf buds) from each nectarine cultivar were used. Shoots were cut into small sections, ground into homogenate in 10% trichloroacetic acid solution and subjected to malondialdehyde (MDA) content determination using the Thiobarbituric acid method [29]. For the determination of proline (PRO) content, 0.5 g shoot sections were added into 5 mL 3% sulfosalicylic acid solution, kept in boiling water for 10 min for PRO extraction, and detected using acid ninhydrin method [30]. To determine the soluble sugar (SS) content, 0.1 g of shoot sections were added to 10 mL 80% ethanol, extracted in a water bath at 80 °C for 30 min, and then measured using the Anthrone-Sulfuric acid colorimetry method [31]. To determine the soluble protein (SP) content, 0.1 g shoot sections were ground into homogenate in precooled (4 °C) 0.1 mol/L phosphate buffer solution (PBS, pH = 7.8) and measured using the Coomassie brilliant blue G-250 method [32]. For enzyme activities detection, 3 g of shoot sections were added into 4 mL of precooled 50 mmol/L PBS (pH7.8), ground into homogenate, centrifuged at 10,000 rpm for 20 min to collect the supernatant, which was then used as crude enzyme solution for the determination of SOD, POD and CAT activity by using nitrogen blue tetrazole photoreduction method, guaiacol method and UV absorption method [33], respectively. The contents of GA_3_, IAA, and ABA in nectarine shoots were determined by using corresponding assay kit produced by Shanghai Enzyme-linked Biotechnology Company (Shanghai, China). For each parameter, at least three replicates were measured. 

### 2.6. Statistical Analyses

All of the data obtained from our study was calculated using SPSS (Version 27.0, IBM, Armonk, NY, USA) and presented as the mean ± standard deviation of replicates. For the variance and difference significance analysis of the data from the five nectarine cultivars at six timepoints, SPSS 27.0 was applied using Duncan’s method at *p* < 0.05 and *p* < 0.01 levels. The OriginPro 2021 software (QriginLab Corporation, Northampton, MA, USA) and the Canoco software (Version 5.0, Microcomputer Power Corporation, Ithaca, NY, USA) was used for principal component analyses (PCA) and redundancy analysis (RDA) of all of the cold resistance related parameters of the five nectarine cultivars at six timepoints during overwintering, respectively.

## 3. Results

### 3.1. The LT_50_ Values of the Five Nectarine Cultivars

As shown in Table 1, the R^2^ values for the LT_50_ Logistic equations of the five nectarine cultivars were all more than 0.9, indicating that these Logistic equations were very suitable for these cultivars and very believable. The LT_50_ values of the five nectarine cultivars ranged from −31.33 °C to −22.32 °C, and in the order of NL, LP, QH, LY and ZY from the lowest to the highest.

### 3.2. Field Cold Resistance Evaluation Results for the Five Nectarine Cultivars

**Figure 2:**
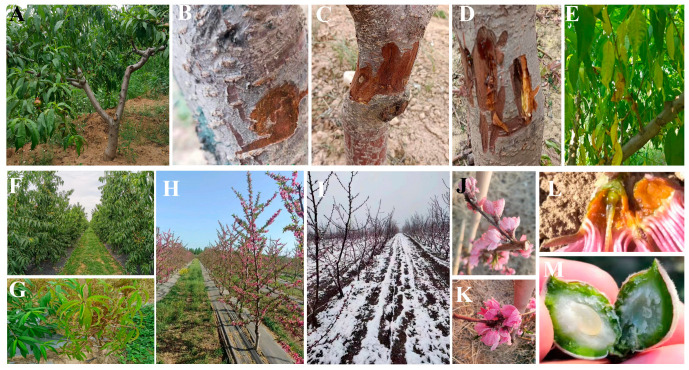
Cold injuries found in nectarine tree trunks, leaves, flowers, and fruits of nectarine trees during natural overwintering. (**A**): healthy control plants without cold injury; (**B**): Grade 1 cold injured trunk (slightly injured trunk with slight browning of xylem); (**C**): Grade 2 cold injured trunk (injured branches and trunk with small cracks); (**D**): Grade 3 cold injured trunk (branches frozen to death, trunk with obvious cracks and colloid outflow); (**E**): leaf wilting; (**F**): normal plant leaves; (**G**): leaf yellowing; (**H**): nectarine flowering condition before freezing; (**I**): flower buds fall off after freezing; (**J**): frozen flowers and flower buds; (**K**,**L**): withered and frozen flowers; (**M**): ice crystals in fruit.

After field investigation, several kinds of cold injuries were observed in nectarine trees, such as no obvious symptom (Figure 2A), slight frostbite in the trunk and slight browning of the xylem (Figure 2B), frostbite in the branches and small cracks in the trunk (Figure 2C), frozen dead branches, and obvious cracks in the trunk with colloid outflow (Figure 2D), wilting and yellow leaves (Figure 2E–G), flower shedding (Figure 2H,I), frozen flower buds, and wilted and gum flowing flowers (Figure 2J–2L). Moreover, ice crystals can be observed in some nectarine fruits (Figure 2M).

According to the symptoms, nectarine trees were categorized into four grades. The proportion of Grade 0 plants of NL (66.67%) and LP (63.33%) was both higher than 60%, followed by QH (60%). The proportion of Grade 0 plants for ZY and LY was 46.67% and 40% (Table 2), respectively. The Grade 1 plants of LP, LY, NL, ZY and QH accounted for 26.67%, 20%, 30%, 16.67% and 26.67%, respectively (Table 2). The proportions of Grade 2 plants for NL, LP and QH were all less than 10%, which were 3.33%, 6.67% and 6.67%, respectively. However, the proportion of Grade 2 plants for LY and ZY was more than 20%, which were 23.33% and 26.67% (Table 2), respectively. It is worth noting that there were 0, one and two Grade 3 plants for NL, LP and QH, respectively. But 16.67% and 10% of the LY and ZY plants were categorized into Grade 3, respectively (Table 2). According to the freezing damage index, it was found that the order of the five cultivars from low to high was NL < LP < QH < ZY < LY. 

### 3.3. The RW Content and RE Changes of the Five Nectarine Cultivars during Overwintering

**Figure 3:**
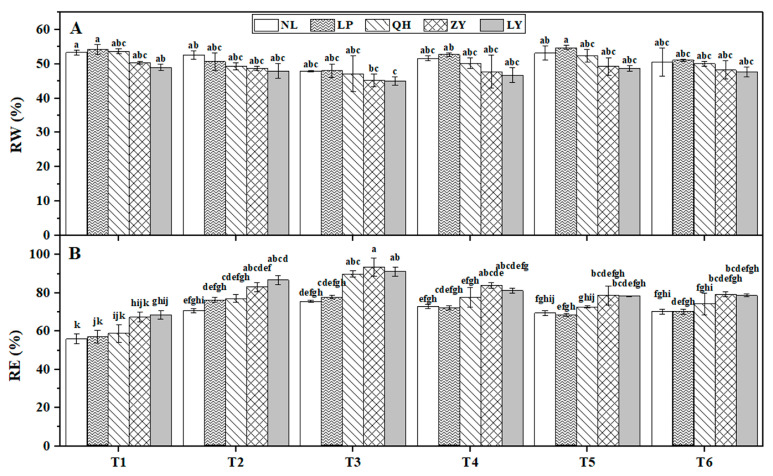
Dynamic changes of relative water (RW) content (**A**) and relative electric conductivity (RE) (**B**) of nectarine varieties at six timepoints (T1–T6) during overwintering. Different lowercase letters above bars represent significant difference at *p* < 0.05 level.

The RW contents (Figure 3A) and RE (Figure 3B) in shoots of the five nectarine cultivars at six timepoints during natural overwintering were measured. Results showed that the change pattern of RW contents of all cultivars was ‘fall (from T1 to T3)-rise (T4 and T5)-fall (T6)’, which was opposite with the change patterns of RE. Although there was no significant RW content difference among cultivars at the same timepoint, the RW contents of LY and ZY were lower than that of the other three cultivars all of the time. However, their RE values were higher or even significantly higher than other cultivars.

Compared to T1, slow temperature drop (9 °C gradual drop of the lowest temperature in a day (LT) in one month from T1 to T2) lead to no significant RW content reduction, but significant RE increasement for all cultivars at T2. Interestingly, fast and sudden temperature drop at T6 led to a more than 5% RW content reduction for both LP and NL (7.18% and 5.29%, respectively) compared to T5, while the reduction ratio for QH, LY and ZY was only approximately 4.66%, 2.10%, and 2.07%, respectively. These results suggested that the water content adaptabilities to low temperature of LP and NL were much stronger than other three cultivars, which might be closely related to their high cold resistance. 

### 3.4. Osmoregulatory Substances Contents Changes of the Five Nectarine Cultivars during Natural Overwintering

The contents of PRO, MDA, SS and SP in shoots of the five nectarine cultivars were measured (Table 3). Results showed that they all exhibited a ‘rise-fall’ trend during T1–T5 period, and then rise slightly at T6. At T3, the PRO content of LP peaked and was significantly higher than that of LY and ZY. The MDA content of all nectarine cultivars also peaked at T3. Since T4, the MDA contents of all nectarine cultivars decreased gradually with the temperature rising. It is worth noting that the PRO contents of NL and LP were higher, but their MDA contents were lower than other cultivars at all timepoints. The SS content of these five nectarine cultivars followed the order of NL > LP > QH > LY > ZY at most timepoints, and the SS contents of NL and LP were significantly higher than that of LY and ZY at T3. The SP content increased significantly at T2 and reached the highest level at T3. In addition, the SP contents of LP and NL were significantly higher than that of LY and ZY at T1, and the SP content of LP was significantly higher than that of LY and ZY at T3.

### 3.5. Changes of Antioxidant Enzyme Activities in Shoots of the Five Nectarine Cultivars

The SOD and POD activities of all cultivars showed a ‘rise-fall-rise’ trend during T1–T6, while their CAT activity exhibited an opposite trend (Table 4). The SOD activities of LP and NL were both higher than that of the other three cultivars at all of the six timepoints during overwintering, and the SOD activities of LP and NL were significantly higher than that of LY and ZY at T1–T4. The POD activity gradually increased with the decrease of temperature, and reached the highest at T3. At all of the six timepoints, the POD activities of LY and ZY were lower than the other three cultivars. CAT activity decreased with the decreasing of temperature, and the lowest CAT activity of all cultivars were found at T3. At T4 and T5, the CAT activity increased slightly, and the CAT activities of all cultivars declined at T6. Moreover, the CAT activity of LY was found to be significantly higher than LP and NL at both T2 and T3.

### 3.6. Changes of Phytohormones Accumulations in Shoots of the Five Nectarine Cultivars

As shown in Table 5, the GA_3_ content in shoots of all nectarine cultivars exhibited a ‘rise-fall’ trend during T1-T5, and rose slightly at T6. At T2, the GA_3_ contents of LP, NL and QH were significantly lower than that of LY. The IAA content of all nectarine cultivars also exhibited a ‘fall-rise-fall’ trend with the lowest levels at T3. At T4, the contents of GA_3_ and IAA in nectarine shoots gradually increased. After the temperature drop at T6, the contents of GA_3_ and IAA decreased slightly. The ABA contents of all of the five nectarine cultivars increased during T1-T3 and peaked at T3, decreased at T4 and T5, and slightly increased at T6. At T1, the ABA contents of LP and NL were significantly higher than that of LY and ZY. At T3, the ABA content of LP was the highest and was significantly higher than that of ZY. The change trend of ABA/GA_3_ was similar to that of ABA content. At T1, the ABA/GA_3_ value of LP was significantly higher than LY and ZY. At T2, the ABA/GA_3_ value of QH was significantly higher than ZY. At T3, and the ABA/GA_3_ value of LP was significantly higher than ZY. 

### 3.7. Correlation Analysis Results of Cold Resistance Related Parameters

**Figure 4:**
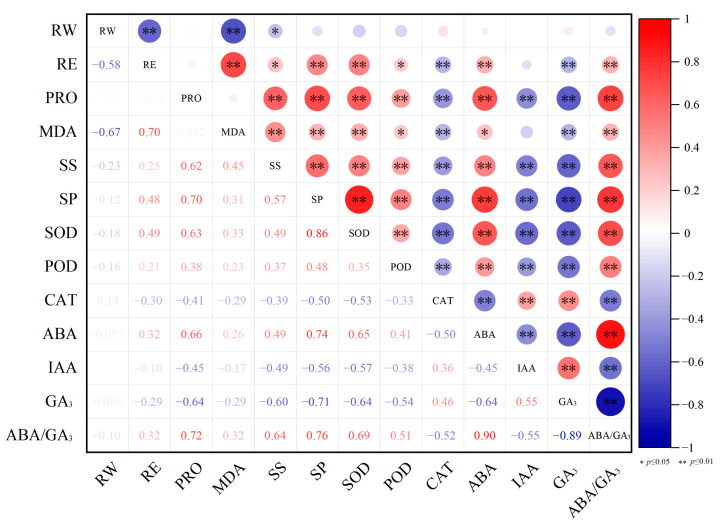
Correlation analysis results of cold resistance related parameters in nectarine cultivars. ‘*’ and ‘**’ indicates significant and very significant correlation, respectively.

SPSS software (Version 27.0) was used to analyze the correlations among cold resistance related parameters (Figure 4). RE was found to be very significantly negatively correlated with RW and GA_3_ contents and CAT activity, very significantly positively correlated with MDA, SP, SOD and ABA contents and ABA/GA_3_, and significantly positively correlated with SS content and POD activity. Significant correlations were also identified among other parameters. For example, significant or very significant positive correlations were identified among contents of PRO, MDA, SS, SP and ABA, activities of SOD and POD, and ABA/GA_3_; significant positive correlations were found among CAT activity, and IAA and GA_3_ contents. Moreover, the RW content was identified to be very significantly negatively correlated with RE and MDA content, and significantly negatively correlated with SS content.

### 3.8. Principal Component Analysis (PCA) Results

**Figure 5:**
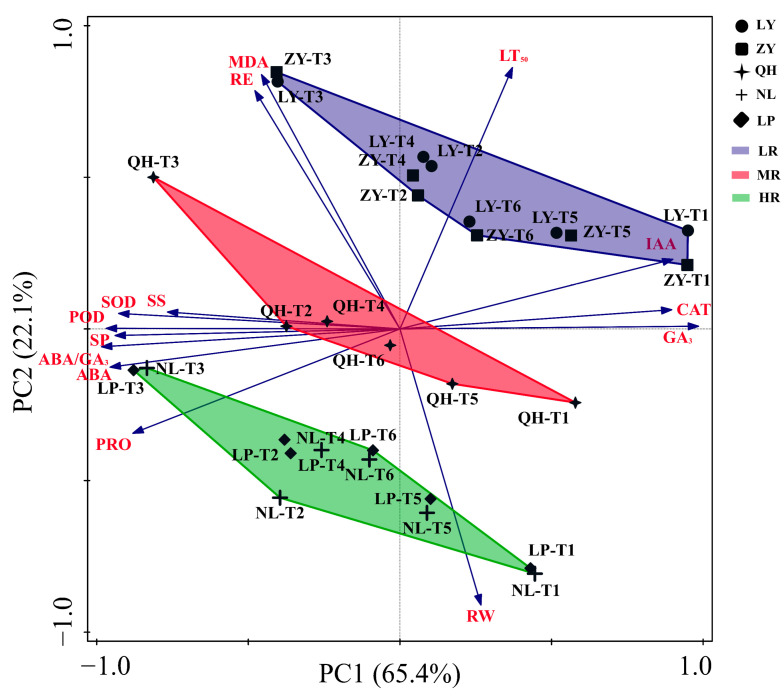
PCA analysis results of the cold resistance related parameters in shoots of different nectarine cultivars. PC: principal component; LR: Low-cold resistant; MR: moderate-cold resistant; HR: High-cold resistant.

Based on the LT_50_ and other thirteen cold resistance related parameters determined in this study, PCA was performed. As shown in Figure 5, the contribution rate of PC1 and PC2 is about 65.4% and 22.1%, respectively. The results of PCA analysis can successfully categorize these five nectarine cultivars into three groups: the high resistant group (HR, including NL and LP) located in the positive PC2 region, the moderate resistant group (MR, including QH) located near the origin, and the low resistant group (LR, including ZY and LY) located in the negative PC2 region. LT_50_ is widely regarded as one of the most important indicators of plant cold resistance [34]. Through PCA analysis, we found that RE, MDA, IAA and GA_3_ contents, and CAT activity (positively correlated with LR cultivars) were positively correlated with LT_50_, while SOD and POD activities, and PRO, SS, ABA and RW contents (positively correlated with HR cultivars) were negatively correlated with LT_50_. 

### 3.9. Redundancy Analysis Results

**Figure 6:**
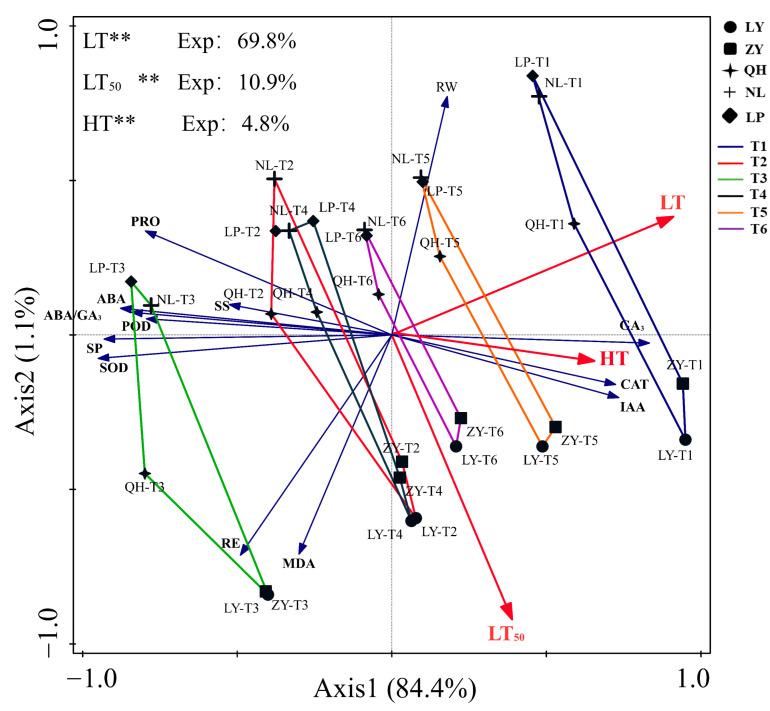
Redundancy analysis results for cold related parameters of nectarine cultivars. Exp: explanation ratio. ‘**’ represents statistically significant factor.

RDA was conducted to reveal the impacts of LT, HT and LT_50_ on cold resistance related parameters. Results showed that the axis1 could explain 84.4% of the variables (Figure 6). LT was identified to be the most significant influential factor with explanation rate of 69.8%, followed by LT_50_ (10.9%), and HT (4.8%). LT was positively correlated with GA_3_, IAA and RW contents and CAT activity, but negatively correlated with SOD and POD activities, PRO, SP, SS and ABA contents and ABA/GA_3_. LT_50_ was positively correlated with RE, MDA, IAA and GA_3_ contents and CAT activity, but negatively correlated with POD and SOD activities, and PRO, SP, SS, ABA and RW contents. In addition, we found that IAA and GA_3_ contents and CAT activity all increased with the rise of LT. At T1, IAA and GA_3_ contents and CAT activity were positively correlated with LR cultivars, while the RW content was positively correlated with HR cultivars. At T3, the activities of POD and SOD, contents of PRO, SP, SS and ABA, and ABA/GA_3_ were positively correlated with HR cultivars (NL and LP), but RE and MDA content were positively correlated with LR cultivars (ZY and LY).

## 4. Discussion

In this study, by determining LT_50_, investigating freezing damages in field and measuring the cold resistance related physio-biochemical parameters, we evaluated and compared the cold resistance of the five main nectarine cultivars in Shanxi Province of China. LT_50_ can directly reflect the cold resistance of plants [34,35]. Our study revealed that the LT_50_ of NL, LP, QH, LY and ZY is −31.33 °C, −29.50 °C, −25.09 °C, −23.25 °C and −22.32 °C, respectively. Consistently, our PCA analysis based on physio-biochemical parameters of these five nectarine cultivars at six timepoints during natural overwintering successfully categorized them into HR (NL and LP), MR (QH) and LR (LY and ZY) groups, indicating that comprehensive analysis of these parameters serves well to illustrate the cold resistance of nectarine germplasm resources. Moreover, our study found that:

### 4.1. High Cold Resistant Nectarine Cultivars Have Higher RW, PRO, SS and SP Contents and Stronger Water and Osmoregulatory Substances Adaptabilities to Low Temperature Compared to Low Cold Resistant Ones

Under low temperature stress, plants would decrease water content and increase osmoregulatory substances accumulations in cells to improve the solute concentration of cytoplasm, thereby reducing the freezing point and alleviating the mechanical damages caused by ice crystals [36]. It was reported that the cold resistance of grapes was close related with their tissue water content loss abilities [37]. Ouyang et al. [38] compared the cold resistance of four rose varieties and found that low cold resistant ‘Abraham Darby’ and ‘Chandos Beauty’ varieties had lower RW content. In this study, we found that the RW contents in shoots of HR cultivars (NL and LP) were significantly higher, and the reduction ratios of their RW contents after low temperature especially fast temperature drop were larger than that of the other three cultivars, suggesting that higher RW content and stronger water loss ability were closely related to their high cold resistance. 

Accumulations of SS, SP, PRO, and some other osmoregulatory substances during natural overwintering were important for the plant defense responses to low temperature [39,40,41,42]. Under low temperature condition, the SS content of apple rootstock significantly increased while the MDA content significantly decreased [40]. The changes of SS, PRO and SP contents of cold-resistant sweet pepper varieties were stronger than these of cold susceptible varieties under low temperature condition [41]. Ying et al. [42] found that the cold resistance of grapevine shoots was close correlated with the SS content in aboveground part, and the contents of SS, SP, PRO, and some other osmoregulatory substances in the aboveground parts increased during natural overwintering. In this study, the contents of PRO, SS and SP in shoots of LR nectarine cultivars were found to be lower than MR and HR cultivars at all timepoints during overwintering, suggesting that the high contents of these three osmoregulatory substances contributed positively to the cold resistance of nectarine. In addition to them, MDA can also be used as an important index for the cold resistance evaluation of plants [17] and its content was generally negatively correlated with cold resistance [43]. Consistently, our study found that the MDA contents of HR cultivars were lower than MR and LR cultivars.

Besides the differences in the basal contents of osmoregulatory substances among different cultivars, the adaptabilities of these substances in response to low temperature also varied [41,44]. In fig (*Ficus carica* L.), the upregulation ratio of SS in the cold resistant variety ‘Atabaki’ was much larger than cold susceptible varieties [45]. In this study, the contents of MDA, PRO, SS and SP of HR cultivars (NL and LP) increased with decreasing temperature, and their change amplitudes were all larger than other cultivars. It can be concluded that the contents of osmotic regulatory substances and their adaptabilities in response to low temperature are positively related to the cold resistance of nectarines.

RE is regarded as one of the important indicators of plant cold resistance [17,46]. The RE of hazelnut varieties with high cold resistance was lower than cold susceptible varieties [47]. Similarly, our study revealed that the RE values of high cold resistant NL and LP cultivars were lower and their RE upregulations after low temperature were milder than LR cultivars. Moreover, through PCA analysis, we found that the RE of nectarine was very significantly negatively correlated with RW content, very significantly positively correlated with MDA and SP contents, and significantly positively correlated with SS content, which again indicated that the higher basal contents and much stronger RW, PRO, SS and SP adaptabilities in response to low temperature of HR cultivars contribute greatly to their high cold resistance.

### 4.2. High Cold Resistant Nectarine Cultivars Are of Higher SOD and POD Activities but Lower CAT Ability, and IAA and GA_3_ Contents

Antioxidant enzyme activities have been widely confirmed to be closely related with plant cold resistance. The SOD and POD activities of high cold resistant ‘Yuanlin’ and ‘Wen 185’ walnut varieties were reported to be higher than those of the cold susceptible ‘Longpuxiang 2’ variety [34]. In this study, the SOD and POD activities of HR cultivars were found to be significantly higher than those of the other three cultivars, indicating that higher SOD and POD activities were positively related to the cold resistance of nectarines. It was reported that the activities of POD, CAT, and SOD in citrus branches increased at 0 °C, and the activities of POD and SOD continued to increase at −6 °C [48]. Fan et al. [49] studied the cold resistance of *Zanthoxylum armatum* and found that SOD and POD activities increased while CAT activity decreased with the decrease of temperature. In this study, the CAT activity was found to be positively correlated with temperature, which might be related the very low temperature (<0 °C) during overwintering [8,50]. 

The basal contents and changes of endogenous phytohormones also contribute greatly to the plant cold resistance ability. Under low temperature stress, the ABA accumulation increased, while the contents of auxin and gibberellin decreased in young wheat ears [51]. The ABA content of wheat increased rapidly after cold stress, and high cold resistant varieties were found to have higher ABA content [52]. In kiwifruit, the GA_3_ content in branches of low cold tolerant varieties was reported to be higher, and the GA_3_ content decreased after low temperature stress [48]. Similarly, the GA_3_ content in rice anther decreased in response to low temperature [53]. The contents of GA_3_ and IAA of both cold susceptible and cold tolerant varieties reduced under low temperature stress (9 °C) [54]. In this study, the contents of IAA and GA_3_ in in shoots of HR cultivars were found to be lower than that of MR and LR cultivars, indicating that they were negatively correlated with the cold resistance of nectarines. In addition, we found that the ABA contents in HR and MR cultivars were always higher than LR cultivars. Furthermore, the ABA/GA_3_ value decreased with temperature, indicating that there was an antagonism between ABA and GA_3_ under low temperature stress. 

## 5. Conclusions

**Figure 7:**
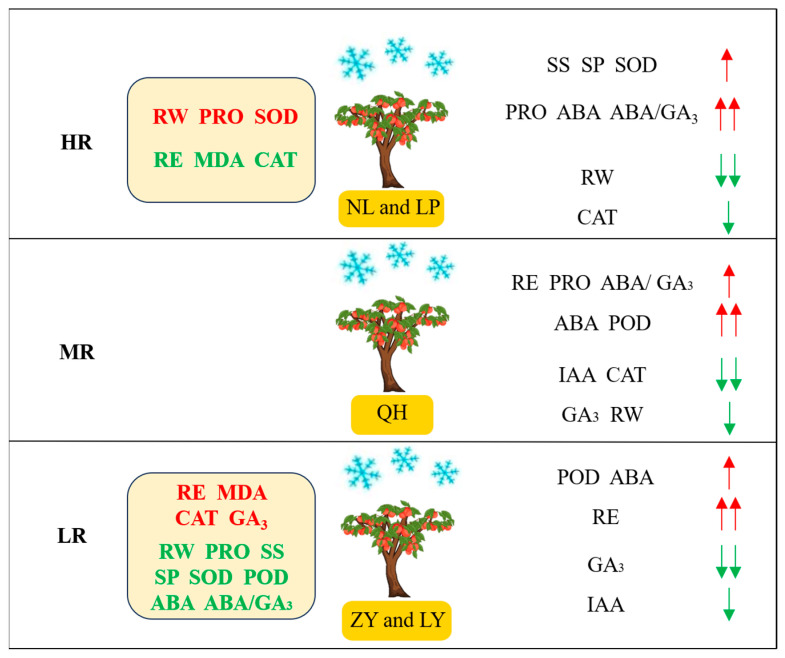
The physio-biochemical mechanism underlying the cold resistance of nectarine cultivars. HR: high cold resistant; MR: Moderate cold resistant; LR: low cold resistant. Parameters in red and green represent that their values were the highest and the lowest, respectively. Red and green arrows represent upregulation and downregulation after low temperature, respectively.

In this study, we evaluated and compared the cold resistance of five main nectarine cultivars in Shanxi province of China and explored the physio-biochemical mechanism underlying their cold resistance variations (Figure 7). Our study successfully categorized them into HR (NL and LP), MR (QH) and LR (LY and ZY) groups. The RW and PRO contents, SOD activities of HR cultivars were higher but their RE, MDA content and CAT activity were lower than MR and LR cultivars. The RE, MDA and GA_3_ contents and CAT activity of LR cultivars were higher than HR and MR cultivars. In addition, the adaptabilities of some parameters, such as RW, IAA, RE and so on, also differed greatly among HR, MR and LR cultivars. Contents of PRO, SP, SS and RW were found to be positively correlated with HR cultivars, while contents of MDA, IAA, GA_3_ were positively correlated with LR cultivars, indicating that they were of great potential to be used for the cold resistance evaluation of nectarine germplasm resources. Our study will be helpful for the comprehensive cold resistance evaluations of nectarine germplasm resources and can provide a basis for the ‘high cold resistance’ targeted new nectarine variety selection and breeding.

## Figures and Tables

**Table 1:** Logistic equation and LT_50_ value for each nectarine cultivar.

Cultivar	Relative Electrolytic Leakage Rate/%						Regression Equation	R^2^	LT_50_/°C
	0 °C	−10 °C	−15 °C	−20 °C	−25 °C	−30 °C			
NL	17.81	27.46	31.36	32.39	38.83	53.26	$Y=100/(1+4.6780e^{-0.0493x})$	0.9356	−31.33
LP	20.07	25.46	30.46	32.28	46.83	54.26	$Y=100/(1+4.6154e^{-0.0520x})$	0.9430	−29.50
QH	19.07	26.46	32.46	39.28	47.83	62.26	$Y=100/(1+4.8715e^{-0.0628x})$	0.9628	−25.09
LY	27.00	35.62	39.05	42.25	50.26	65.64	$Y=100/(1+3.2710e^{-0.0510x})$	0.9370	−23.25
ZY	24.00	35.62	39.05	42.25	48.26	63.64	$Y=100/(1+3.0283e^{-0.0496x})$	0.9084	−22.32

**Table 2:** Cold resistance investigation and classification results for the five nectarine cultivars. Different lowercase letters in the last row indicate significant difference of freezing damage index. The higher the freezing damage index, the lower the cold resistance.

Cultivar	Total No.	Grade 0		Grade 1		Grade 2		Grade 3		Freezing Damage Index
		No.	Rate %	No.	Rate %	No.	Rate %	No.	Rate %	
NL	30	20	66.67	9	30.00	1	3.33	0	0	0.34 e
LP	30	19	63.33	8	26.67	2	6.67	1	3.30	0.38 d
QH	30	18	60.00	8	26.67	2	6.67	2	6.67	0.40 c
LY	30	12	40.00	6	20.00	7	23.33	5	16.67	0.54 a
ZY	30	14	46.67	5	16.67	8	26.67	3	10.00	0.50 b

**Table 3:** Osmoregulatory substances contents in shoots of the five nectarine cultivars during natural overwintering. PRO: proline; MDA: malondialdehyde; SS: soluble sugar; SP: soluble protein. Different lowercase letters in the same row indicate significant difference at the *p* < 0.05 level.

Time	Cultivar	PRO (μg/g)	MDA (mmol/g)	SS (mg/g)	SP (mg/g)
T1	NL	54.38 ± 2.72 bcdefg	3.61 ± 0.13 de	56.94 ± 0.60 efghij	0.54 ± 0.03 hij
	LP	52.58 ± 2.90 bcdefg	3.37 ± 0.30 e	57.96 ± 1.87 efghi	0.47 ± 0.03 j
	QH	46.63 ± 2.92 defg	4.23 ± 0.13 bcde	58.27 ± 0.83 efghi	0.34 ± 0.01 k
	ZY	35.09 ± 2.99 g	4.67 ± 0.23 bcde	46.35 ± 2.57 fghijk	0.19 ± 0.02 l
	LY	38.90 ± 0.96 fg	5.16 ± 0.28 abcde	48.47 ± 1.00 fghijk	0.21 ± 0.03 l
T2	NL	69.50 ± 2.96 abc	4.72 ± 0.27 bcde	71.18 ± 1.35 de	0.84 ± 0.02 abc
	LP	62.70 ± 1.77 abcde	4.18 ± 0.79 bcde	70.13 ± 0.86 de	0.88 ± 0.01 ab
	QH	61.03 ± 10.83 abcde	5.00 ± 0.25 abcde	70.10 ± 1.66 de	0.84 ± 0.01 abc
	ZY	55.37 ± 1.50 bcdefg	5.80 ± 0.51 abc	60.13 ± 0.13 efgh	0.68 ± 0.02 defg
	LY	48.95 ± 3.52 defg	5.76 ± 0.51 abc	62.36 ± 2.53 ef	0.75 ± 0.02 cdef
T3	NL	70.66 ± 6.74 ab	5.87 ± 0.48 ab	117.37 ± 10.30 a	0.84 ± 0.02 abc
	LP	77.52 ± 4.40 a	5.52 ± 0.53 abcd	104.68 ± 7.81 ab	0.92 ± 0.01 a
	QH	63.56 ± 6.24 abcd	6.57 ± 0.15 a	101.73 ± 4.33 bc	0.89 ± 0.00 ab
	ZY	56.98 ± 4.86 bcdef	6.90 ± 0.35 a	85.71 ± 5.32 d	0.75 ± 0.01 cdef
	LY	52.80 ± 4.25 bcdefg	6.85 ± 0.44 a	87.00 ± 3.16 cd	0.80 ± 0.03 abcd
T4	NL	56.87 ± 1.79 bcdef	4.79 ± 0.48 bcde	71.61 ± 0.95 de	0.78 ± 0.03 bcde
	LP	58.29 ± 4.36 bcdef	4.38 ± 0.39 bcde	56.81 ± 1.92 efghij	0.74 ± 0.03 cdef
	QH	53.27 ± 10.44 bcdefg	5.27 ± 0.35 abcde	60.38 ± 5.45 efg	0.74 ± 0.02 cdef
	ZY	48.34 ± 3.52 defg	5.51 ± 0.26 abcd	54.57 ± 3.66 efghij	0.67 ± 0.02 efg
	LY	47.51 ± 4.09 defg	5.93 ± 0.59 ab	41.61 ± 4.55 hijkl	0.66 ± 0.02 efgh
T5	NL	52.62 ± 2.39 bcdefg	3.73 ± 0.11 cde	45.70 ± 3.55 fghijk	0.64 ± 0.02 fghi
	LP	55.06 ± 6.24 bcdefg	3.94 ± 0.13 bcde	38.65 ± 0.77 jkl	0.67 ± 0.03 efg
	QH	50.21 ± 4.03 cdefg	4.49 ± 0.35 bcde	42.15 ± 2.42 ghijkl	0.64 ± 0.03 fghi
	ZY	44.55 ± 4.79 defg	4.58 ± 0.19 bcde	31.35 ± 2.60 kl	0.54 ± 0.01 ij
	LY	42.03 ± 5.56 efg	4.75 ± 0.42 bcde	25.39 ± 2.16 l	0.51 ± 0.02 j
T6	NL	58.01 ± 0.78 bcdef	4.19 ± 0.35 bcde	53.15 ± 3.98 efghij	0.68 ± 0.05 defg
	LP	60.13 ± 1.44 abcde	3.97 ± 0.49 bcde	39.99 ± 2.07 ijkl	0.72 ± 0.03 cdef
	QH	55.51 ± 2.73 bcdef	4.60 ± 0.28 abcde	45.04 ± 2.88 fghijk	0.67 ± 0.02 efgh
	ZY	49.53 ± 5.82 cdefg	4.80 ± 0.43 bcde	32.33 ± 2.12 kl	0.56 ± 0.04 ghij
	LY	47.80 ± 2.19 defg	5.17 ± 0.50 abcde	31.65 ± 2.99 kl	0.54 ± 0.03 ij

**Table 4:** Changes of antioxidant enzymes activities in shoots of the five nectarine cultivars during natural overwintering process. SOD: superoxide dismutase; POD: peroxidase; CAT: catalase. Different lowercase letters in the same row indicate significant difference at the *p* < 0.05 level.

Time	Cultivar	SOD (U/g FW)	POD (U/g FW)	CAT (U/g FW)
T1	NL	330.00 ± 1.56 efgh	33.73 ± 2.26 abc	47.00 ± 4.79 abc
	LP	325.13 ± 4.71 fgh	35.53 ± 1.74 abc	45.03 ± 4.28 abc
	QH	303.10 ± 12.48 gh	34.18 ± 0.88 abc	51.33 ± 4.68 ab
	ZY	283.50 ± 9.89 h	27.33 ± 1.90 bc	53.33 ± 2.92 a
	LY	298.46 ± 9.74 gh	26.67 ± 3.84 c	54.33 ± 2.34 a
T2	NL	582.62 ± 16.32 ab	45.67 ± 2.08 abc	38.33 ± 4.49 abc
	LP	574.41 ± 12.49 ab	46.00 ± 0.33 abc	36.33 ± 1.75 abc
	QH	563.11 ± 6.00 abc	43.33 ± 4.33 abc	44.00 ± 4.55 abc
	ZY	510.85 ± 13.03 abcd	37.33 ± 4.63 abc	47.33 ± 2.36 abc
	LY	505.43 ± 4.39 abcd	36.33 ± 3.84 abc	49.67 ± 2.47 ab
T3	NL	600.43 ± 17.95 a	50.33 ± 3.76 abc	27.33 ± 0.94 c
	LP	594.91 ± 10.54 a	54.33 ± 0.88 a	26.67 ± 1.65 c
	QH	584.02 ± 20.81 ab	53.33 ± 3.18 ab	30.53 ± 3.63 bc
	ZY	541.04 ± 9.36 abcd	47.00 ± 3.84 abc	34.00 ± 4.09 abc
	LY	529.05 ± 5.82 abcd	46.67 ± 3.61 abc	36.67 ± 4.50 abc
T4	NL	556.62 ± 15.23 abc	47.33 ± 7.24 abc	31.00 ± 3.07 bc
	LP	560.27 ± 8.62 abc	44.00 ± 5.32 abc	32.00 ± 5.86 bc
	QH	532.64 ± 9.63 abcd	47.67 ± 5.87 abc	34.00 ± 8.34 abc
	ZY	453.84 ± 11.09 bcde	44.33 ± 3.29 abc	37.33 ± 1.97 abc
	LY	472.45 ± 25.27 abcd	42.67 ± 4.11 abc	39.00 ± 7.73 abc
T5	NL	492.13 ± 6.94 abcd	39.00 ± 4.61 abc	44.33 ± 3.10 abc
	LP	488.54 ± 76.32 abcd	40.00 ± 4.50 abc	42.67 ± 2.62 abc
	QH	465.12 ± 44.90 abcd	37.33 ± 3.06 abc	46.00 ± 4.26 abc
	ZY	412.17 ± 8.08 defg	31.00 ± 5.15 abc	47.67 ± 5.71 abc
	LY	428.51 ± 61.86 cdef	32.00 ± 8.00 abc	49.85 ± 4.22 ab
T6	NL	508.45 ± 8.48 abcd	41.33 ± 2.52 abc	35.33 ± 2.67 abc
	LP	506.80 ± 22.12 abcd	42.00 ± 4.37 abc	36.00 ± 4.96 abc
	QH	484.65 ± 15.22 abcd	39.67 ± 4.04 abc	39.00 ± 2.13 abc
	ZY	447.27 ± 30.07 bcdef	35.33 ± 2.85 abc	42.67 ± 0.76 abc
	LY	450.63 ± 61.71 bcdef	36.00 ± 3.18 abc	41.33 ± 4.15 abc

**Table 5:** Changes of phytohormone accumulations in the five nectarine cultivars during natural overwintering process. GA_3_: gibberellin acid; IAA: indoleacetic acid; ABA: abscisic acid. Different lowercase letters in the same row indicate significant difference at the *p* < 0.05 level.

Time	Cultivar	GA_3_ (ng/g FW)	IAA (ng/g FW)	ABA (ng/g FW)	ABA/GA_3_
T1	NL	8.07 ± 0.44 abcd	116.09 ± 3.74 a	294.61 ± 8.88 ghij	36.51 ± 3.88 defg
	LP	7.97 ± 0.07 abcd	98.08 ± 3.86 abcde	307.1 ± 13.54 fghi	38.55 ± 0.94 defg
	QH	8.01 ± 0.30 abcd	114.49 ± 6.32 cde	292.09 ± 16.64 ghij	36.49 ± 4.97 defg
	ZY	8.78 ± 0.13 ab	98.78 ± 6.75 abcde	258.80 ± 14.57 ij	29.47 ± 1.90 fg
	LY	9.16 ± 0.38 a	100.60 ± 4.02 abcde	239.41 ± 14.94 j	26.13 ± 5.72 g
sT2	NL	6.75 ± 0.28 bcd	99.45 ± 5.31 abcde	380.58 ± 21.61 abcde	56.39 ± 5.04 abcd
	LP	6.83 ± 0.27 bcd	88.03 ± 8.22 de	374.44 ± 4.65 abcde	54.79 ± 1.28 abcde
	QH	6.48 ± 0.24 cd	101.60 ± 6.70 abcde	394.65 ± 22.85 abcd	60.87 ± 5.38 abc
	ZY	7.46 ± 0.50 abcd	91.26 ± 4.47 ab	328.17 ± 25.29 efgh	44.02 ± 4.68 cdefg
	LY	7.80 ± 0.36 abcd	87.03 ± 6.11 de	338.26 ± 8.45 defgh	43.34 ± 3.08 cdefg
T3	NL	5.96 ± 0.58 d	90.76 ± 2.32 cde	400.78 ± 33.17 abc	67.28 ± 5.86 a
	LP	6.17 ± 0.10 d	86.96 ± 5.83 de	416.96 ± 3.54 a	67.62 ± 0.35 a
	QH	6.02 ± 0.10 d	92.95 ± 0.85 cde	403.73 ± 14.36 ab	67.06 ± 1.37 ab
	ZY	6.72 ± 0.11 bcd	85.54 ± 0.65 de	351.90 ± 31.78 bcdefg	52.36 ± 3.56 abcde
	LY	6.58 ± 0.24 cd	81.07 ± 5.34 e	363.57 ± 9.16 abcdef	55.28 ± 2.17 abcde
T4	NL	6.83 ± 0.58 bcd	105.87 ± 4.43 abcd	369.67 ± 25.99 abcde	54.09 ± 3.41 abcde
	LP	7.07 ± 0.16 abcd	92.29 ± 2.32 cde	377.18 ± 18.98 abcde	53.37 ± 3.03 abcde
	QH	6.96 ± 0.69 bcd	104.74 ± 5.61 abcd	362.07 ± 21.00 abcdef	52.00 ± 5.51 abcde
	ZY	7.34 ± 0.15 abcd	88.85 ± 7.29 de	334.77 ± 25.03 efgh	45.64 ± 3.70 bcdefg
	LY	7.36 ± 0.37 abcd	90.57 ± 3.51 cde	329.9 ± 14.21 efgh	44.81 ± 5.21 bcdefg
T5	NL	7.36 ± 0.72 abcd	110.22 ± 9.64 abc	347.47 ± 14.81 bcdefg	47.22 ± 7.00 abcdefg
	LP	7.48 ± 0.09 abcd	102.10 ± 4.09 abcde	356.41 ± 16.04 bcdef	47.63 ± 1.43 abcdefg
	QH	7.59 ± 0.41 abcd	105.74 ± 6.66 abcd	343.15 ± 34.50 cdefg	45.18 ± 5.90 bcdefg
	ZY	8.36 ± 0.44 abc	95.70 ± 10.68 abcde	282.71 ± 29.51 hij	33.83 ± 8.62 efg
	LY	8.12 ± 0.10 abcd	94.24 ± 2.31 bcde	293.47 ± 20.74 ghij	36.16 ± 2.04 defg
T6	NL	7.25 ± 0.63 abcd	104.35 ± 7.05 abcd	362.44 ± 26.91 abcdef	50.02 ± 4.34 abcdef
	LP	7.25 ± 0.29 abcd	95.59 ± 5.88 abcde	359.77 ± 12.64 abcdef	49.61 ± 3.63 abcdef
	QH	7.14 ± 0.78 abcd	103.45 ± 6.24 abcd	357.50 ± 23.65 abcdef	50.04 ± 6.79 abcdef
	ZY	7.76 ± 0.32 abcd	97.77 ± 8.63 abcde	338.74 ± 41.90 defgh	43.68 ± 4.73 cdefg
	LY	7.78 ± 0.40 abcd	93.64 ± 10.42 bcde	342.41 ± 27.77 cdefgh	44.04 ± 3.10 cdefg

## Data Availability

Data are contained within the article.
